# Torsional flexibility in zinc–benzenedicarboxylate metal–organic frameworks†

**DOI:** 10.1039/d3ce01078c

**Published:** 2023-12-29

**Authors:** Emily G. Meekel, Thomas C. Nicholas, Ben Slater, Andrew L. Goodwin

**Affiliations:** a Inorganic Chemistry Laboratory South Parks Road Oxford OX1 3QR UK andrew.goodwin@chem.ox.ac.uk; b Department of Chemistry, University College London 20 Gordon Street London WC1H 0AJ UK

## Abstract

We explore the role and nature of torsional flexibility of carboxylate–benzene links in the structural chemistry of metal–organic frameworks (MOFs) based on Zn and benzenedicarboxlyate (bdc) linkers. A particular motivation is to understand the extent to which such flexibility is important in stabilising the unusual topologically aperiodic phase known as TRUMOF-1. We compare the torsion angle distributions of TRUMOF-1 models with those for crystalline Zn/1,3-bdc MOFs, including a number of new materials whose structures we report here. We find that both periodic and aperiodic Zn/1,3-bdc MOFs sample a similar range of torsion angles, and hence the formation of TRUMOF-1 does not require any additional flexibility beyond that already evident in chemically-related crystalline phases. Comparison with Zn/1,4-bdc MOFs does show, however, that the lower symmetry of the 1,3-bdc linker allows access to a broader range of torsion angles, reflecting a greater flexibility of this linker.

## Abstract 2

An investigation of the impact of torsional flexibility of benzene dicarboxylate linkers in zinc metal–organic frameworks.

## Graphical abstract

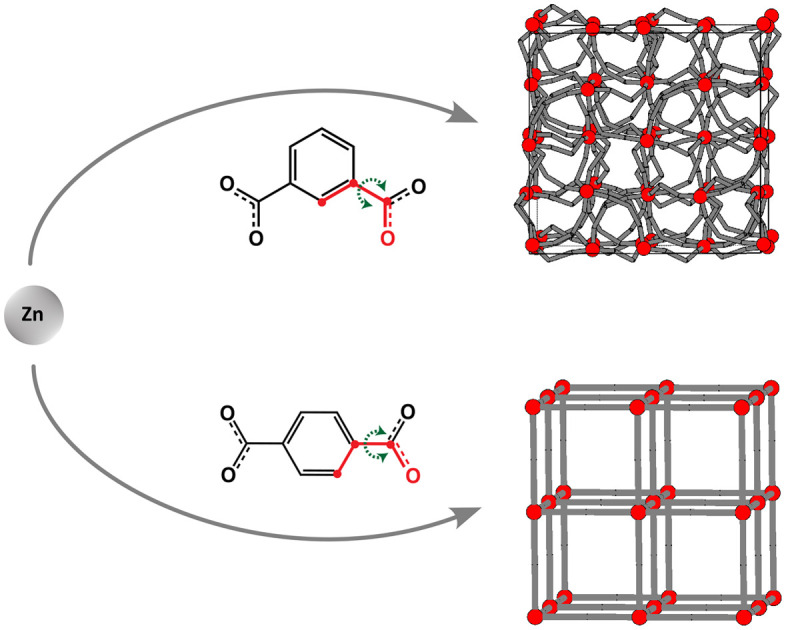

## 1 Introduction

**Fig. 1:**
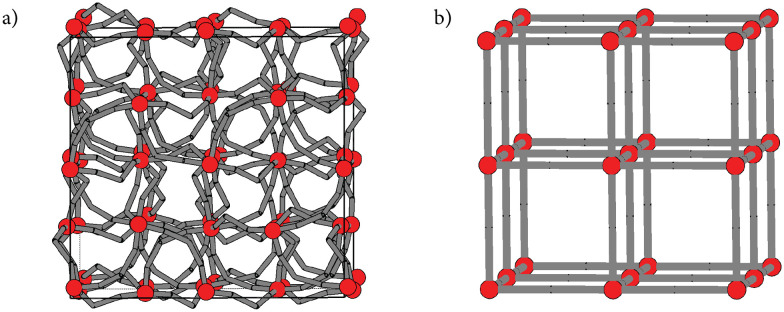
Schematic representations of the topologies of (a) a 2 × 2 × 2 approximant of the TRUMOF-1 structure and (b) MOF-5. In both cases, red circles represent octahedrally-coordinated OZn_4_ clusters. The grey sticks indicate the connectivity formed as clusters are connected using (a) 1,3-bdc and (b) 1,4-bdc linkers.

Metal–organic frameworks (MOFs) assembled from zinc and benzenedicarboxylates are a broad family with potential applications in gas storage,^1^ separation,^2,3^ and chemical sensing.^4–6^ In a recent study, we reported that the particular system OZn_4_(1,3-bdc)_3_ (1,3-bdc = 1,3-benzenedicarboxylic acid), known as TRUMOF-1, adopts a topologically aperiodic network structure, which can be understood as an atomic-scale realisation of a complex three-dimensional Truchet tiling.^7^ The structure of TRUMOF-1 consists of octahedrally-coordinated OZn_4_ clusters arranged on a face-centred cubic (fcc) lattice, with 1,3-bdc linkers connecting pairs of neighbouring clusters. Despite a uniform local connectivity, the arrangement of 1,3-bdc linkers is not periodic and the network structure of TRUMOF-1 never repeats [Fig. 1(a)]. The formation of this unusual state—occupying an intermediate regime between conventional crystals and amorphous continuous random networks—appears to be driven by the 1,3-bdc linker, since use of the more symmetric 1,4-bdc isomer leads to formation of the famous (crystalline) MOF-5 system [Fig. 1(b)].^8^

**Fig. 2:**
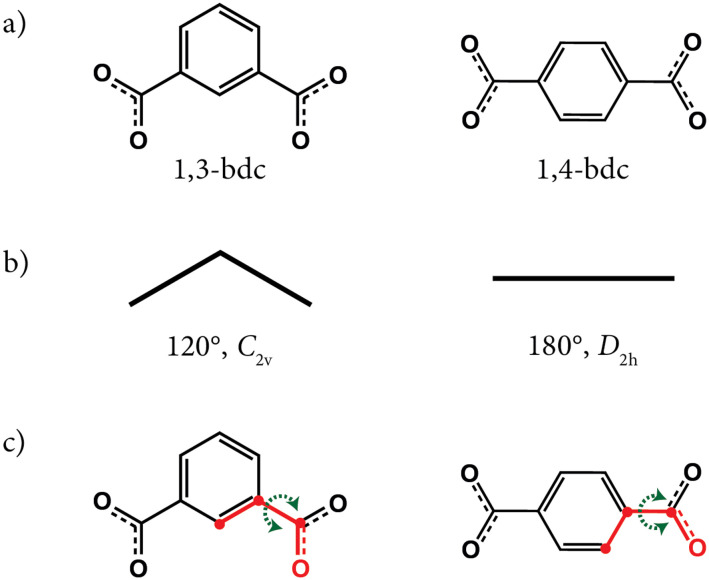
The two linkers 1,3-bdc and 1,4-bdc and their geometric differences: (a) molecular structures; (b) the resulting bent *vs.* linear geometries; and (c) their carboxylate–benzene torsion angle flexibility. For both linkers, the dihedral angle corresponding to the torsion angle is indicated in (c) for one of the carboxylates by highlighting the relevant C–C–C–O bonds and atoms in red.

The two linkers 1,3-bdc and 1,4-bdc differ not only in terms of their binding angles [Fig. 2(a and b)] but also in terms of the barrier to torsional flexing around the benzene–carboxylate link [Fig. 2(c)]—the latter a consequence of differing extents of conjugation.^9^ For example, studies of the rotor behaviour of the central benzene ring in the 1,4-bdc linkers of MOF-5 (ref. 9) are consistent with a large energy barrier to torsional flipping; this barrier is thought to be approximately 50–60 kJ mol^−1^ on the basis of solid-state NMR measurements and DFT calculations.^10–12^ The energetic landscape of 1,3-bdc, on the other hand, is characterised by a much shallower potential well at low rotational angles.^13,14^ As a consequence, the benzene ring of 1,3-bdc may undergo large-amplitude oscillations or librations more easily than that of 1,4-bdc.^15^

An obvious question therefore is whether the unusual structure of TRUMOF-1 is somehow associated with strong torsional distortions of the 1,3-bdc linker in a way not found in conventional crystalline frameworks and/or would not be possible with the ostensibly more rigid 1,4-bdc linker. We approach answering this question in three steps. First, we characterise the distribution of torsional angles observed in approximant structural models of TRUMOF-1 obtained using geometry optimisation with density functional theory (DFT) calculations. We then report a number of new crystalline Zn/1,3-bdc framework structures, and carry out a similar geometric analysis of torsional variability amongst these new materials. We also include in our analysis the structures of a number of previously-reported Zn/1,3-bdc frameworks. Finally, we contrast our results with a geometric analysis of known Zn/1,4-bdc MOF systems (including MOF-5). Taken together, we show that TRUMOF-1 is not associated with any particularly unusual degree of torsional flexibility, suggesting that the electronic structure of 1,3-bdc is not by itself particularly important in stabilising the Truchet-tile architecture. As a corollary, we find the higher symmetry of 1,4-bdc does indeed constrain torsional degrees of freedom for many Zn/1,4-bdc MOFs, which we argue helps explain why reticular chemistry approaches are more successful for that family than for Zn/1,3-bdc systems.^16^

## 2 Results and discussion

### 2.1 Torsion angle distributions in TRUMOF-1

**Fig. 3:**
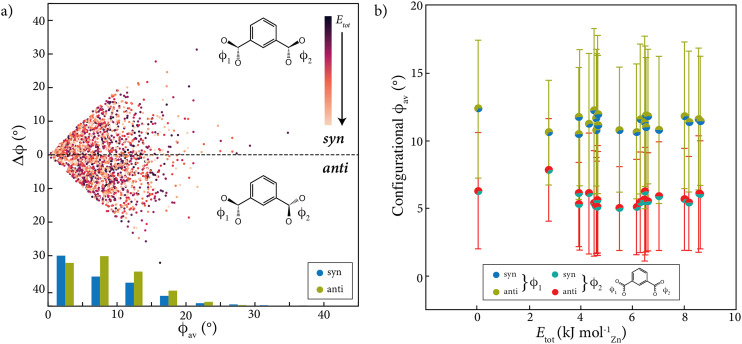
Distribution of 1,3-bdc torsion angle values observed in TRUMOF-1 DFT supercell configurations. (a) Plot showing the distribution of both average torsion angle magnitude, *ϕ*_av_, and difference in torsion angle magnitudes Δ*ϕ* within each 1,3-bdc linker in the various supercells. Linkers with *syn* and *anti* conformations are plotted above and below the central dashed line, respectively. All torsion angle values are coloured according to the DFT energy of the supercell to which they correspond. Histograms are given at the bottom of the graph to demonstrate the ratio of *syn* : *anti* carboxylates within each 5° interval. (b) Graph showing the average values of *ϕ*_1_ and *ϕ*_2_ for each supercell as a function of its relative DFT energy. Individual data points are represented as pie-charts that reflect the ratio of *syn* : *anti* linkers. Note that in order to calculate the average value of *ϕ*_1_ and *ϕ*_2_, the carboxylate with the largest torsion angle was assigned *ϕ*_1_, leaving the smaller torsion angle to be assigned *ϕ*_2_. The standard deviation in torsion angle is represented with error bars.

We started by extracting the torsion angles for each 1,3-bdc linker in the series of 22 DFT-optimised 1 × 1 × 1 and 2 × 2 × 2 approximants^17^ of the TRUMOF-1 supercell configurations reported in ref. 7. This ensemble includes 2134 linkers in total, and hence 4268 torsion angles. Our results are shown in Fig. 3(a). In this representation, the torsional geometry of each linker maps onto a single point. Denoting the two torsion angles for a given linker as *ϕ*_1_ and *ϕ*_2_, then the linker is said to adopt a *syn* geometry if *ϕ*_1_ and *ϕ*_2_ have the same sign, and an *anti* geometry if the signs are opposite. We show our data in terms of the average torsional magnitude1
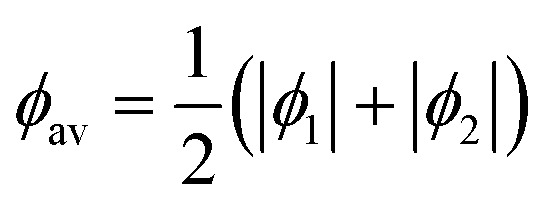
and the torsional asymmetry

2 Δ*ϕ* = ||*ϕ*_1_| − |*ϕ*_2_||,

which captures the difference in torsional angles for a single 1,3-bdc linker. It is a simple geometric constraint that 0 ≤ Δ*ϕ* ≤ 2*ϕ*_av_, which gives rise to the ‘forbidden’ regions of our scatter plot in the top-left and bottom-left corners. We also include in our analysis a histogram of the average torsion angle magnitude, evaluated separately for *syn* and *anti* conformations.

We certainly observe a wide distribution of torsion angle values—the bulk lie between 0 and 20°, and some carboxylates achieve a torsion angle up to 40°. We were curious as to whether the more distorted 1,3-bdc geometries were to be found in configurations with higher DFT energies. Fig. 3(b) shows the average torsion angles within entire configurations as a function of corresponding relative energy; no meaningful correlation is found. This hints towards a shallow energy landscape, which is in agreement with our previous analysis of the DFT configurations.^7^ We also find no correlation between approximant density and torsion angle magnitude (see ESI†). Other geometric descriptors—such as distributions of ZnO_4_ tetrahedral distortions and node–linker–node angles—may therefore account for the energy variation amongst TRUMOF-1 approximants. We anticipate pursuing this point in a future study.

The histograms plotted in Fig. 3(a) show that the number of *syn* linkers gradually decreases with increasing torsion angle, whilst the *anti* linkers increase from the 0–5° to 5–10° range, after which the number gradually decreases. With a distribution of 44% and 56% for the *syn* and *anti* linkers, respectively, there is a slight preference for the latter (see Table S1†). However, there is no meaningful correlation between supercell energy and overall *syn* : *anti* ratio (Fig. 3(b)).

### 2.2 Synthesis of new Zn/1,3-bdc MOFs

In order to understand whether the torsional angle distribution observed for TRUMOF-1 is similar or different to that of crystalline, topologically periodic, Zn/1,3-bdc frameworks, we synthesised a number of such frameworks using the same or related Zn and 1,3-bdc precursors. We included, for example, functionalised 1,3-bdc linkers in order to investigate the application of reticular chemistry to TRUMOF-1 and the overall Zn/1,3-bdc phase space. In the MOF field, reticular chemistry is an important concept to systematically design MOFs of the same topology, but with a tailored functionality and/or pore size.^16^ The linker 1,4-bdc and its variants form a number of well-established isoreticular families. The canonical example is the IRMOF series,^1^ which includes MOF-5, but well-known 1,4-bdc-containing MOFs such as UiO-66,^18^ MIL-53 (ref. 19 and 20) and MIL-88B^21^ have all been used as the basis for isoreticular MOF analogues with functionalised variations of 1,4-bdc.

TRUMOF-1 was originally obtained through a solvothermal synthesis in DMF at 110 °C for 14 h.^7^ We found that changing the synthetic parameters such as the reaction time or temperature did not lead to single crystals of a phase other than TRUMOF-1. However, changing the reaction solvent and/or incorporating functionalised 1,3-bdc resulted in the formation of three new crystalline phases, which we call MOX-2, MOX-3, and MOX-4(X). Internally, we refer to TRUMOF-1 as MOX-1, the first member of this same series.

#### MOX-2

To obtain Zn/1,3-bdc phases other than TRUMOF-1, we repeated the synthesis of TRUMOF-1 in a range of different solvents—namely, ethyl acetate, THF, DMSO, chloroform, ethanol, DCM, hexane, toluene, and acetone. The reactions in THF, ethanol and acetone resulted in a powder of an unidentified phase, whereas the reactions performed in chloroform, DCM and hexane did not result in a product; only starting material was detected. Likewise, the reaction in DMSO resulted in a clear solution with no solid product at all. However, the reaction in ethyl acetate resulted in a new Zn/1,3-bdc MOF, which we call MOX-2.

**Fig. 4:**
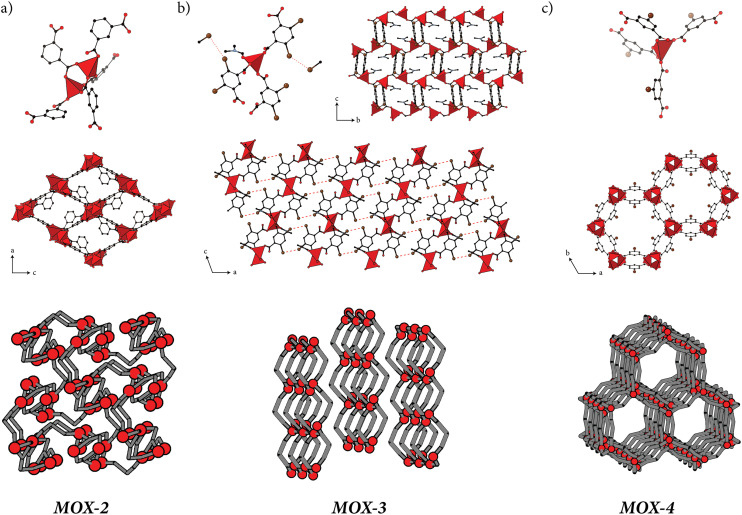
Representations of the crystal structures for the new Zn/1,3-bdc MOFs described in this study. (a) MOX-2 basic building block (top), resulting wine-rack structure viewed down the crystallographic *b*-axis (middle), and topological representation (bottom). Zn coordination environments shown as red polyhedra, and 1,3-bdc linkers shown in ball-and-stick representation. Solvent omitted for clarity. (b) MOX-3 basic building block (top left) and the extended crystal structure viewed down the crystallographic *a*- (top right) and *b*-axes (middle). A topological representation is given at the bottom. (c) MOX-4 basic building block (top), representation of the crystal structure, viewed down the crystallographic *c* axis (middle), and topological representation (bottom). Colour scheme: Zn tetrahedra = red, oxygen = red, carbon = black, nitrogen = blue, Br, Cl, I = brown. Hydrogen atoms are omitted for clarity.

Representations of the structural building unit and crystal structure of MOX-2 are given in Fig. 4(a); full crystallographic details are given in the ESI.† In this structure, pairs of Zn-centred tetrahedra are connected by 1,3-bdc linkers to form a wine-rack-like framework. A striking feature of MOX-2 is that the framework resembles MIL-53 (ref. 22) when viewed down the crystallographic *b* axis. Yet the topology of MOX-2 is different to that of MIL-53, and is given by the symbol **3,5T25**.^23^ MOX-2 has three unique 1,3-bdc linkers, each with a different value for *ϕ*_1_ and *ϕ*_2_, but all in the *syn* configuration [Table S3†].

#### MOX-3

If one repeats the synthesis of TRUMOF-1 (*i.e.* using DMF as solvent) but replaces 1,3-bdc by functionalised 1,3-bdc analogues, then crystalline products are sometimes formed and sometimes not. For example, using the linkers 5-X-1,3-bdc with X = Br, Cl, I, F, CH_3_ and NO_2_, we could not obtain any stable products; the same was true too for 2-I-1,3-bdc. However, using 4,6-Br_2_-1,3-bdc did (eventually) yield crystals of a new phase, which we call MOX-3. In practice, a crystalline product was not directly obtained after synthesis. Instead, hexagonal disk-shaped ‘crystals’ of a metastable phase were first isolated from the reaction vial. These objects were not fully crystalline: strongly diffuse streaks in their diffraction pattern prevented solution of the average structure [Fig. S1 and S2†]. However, after leaving the crystals in oil for several hours, the crystal morphologies changed, evolving into an oval shape [Fig. S1†]. These transformed objects diffracted as single crystals, and belong to the phase MOX-3 which we proceed to describe.

The fundamental building units and longer-range structure of MOX-3 are illustrated in Fig. 4(b); full crystallographic details are again given in the ESI.† In this structure, isolated ZnO_4_ tetrahedra are connected by 4,6-Br_2_-1,3-bdc linkers to form square-grid sheets (topology **sql**) that then stack along the crystallographic *a* axis. This layering brings into close contact the Br substituents of neighbouring layers, suggesting non-covalent halogen-bond interactions may help stabilise this structure. An obvious possibility for the disordered metastable phase that first forms during synthesis is that its structure is assembled from the same two-dimensional layers as in MOX-3, but then the assembly of these occurs with stacking faults caused by the layers slipping over each other.^24^ On isolating the crystals in oil and allowing sufficient time to equilibrate, the layers would then arrange themselves into a crystalline structure in order to maximise non-covalent halogen-bond interactions. We note that, when viewed along the crystallographic *a* axis, pseudo-hexagonal channels are detected, consistent with the ostensibly hexagonal morphology adopted by the metastable crystals.

From a torsion-angle perspective, MOX-3 is particularly striking. The two carboxylates are rotated to give a *syn* conformation, with one adopting a torsional angle of 30.25°, and the second twisting to 83.56° [Table S5†]. We will come to show that this torsion angle is the largest value we have observed for 1,3-bdc in any Zn-containing MOF, and is presumably a function of the large steric bulk associated with dibromination of the central benzene ring.

#### MOX-4

The final Zn/1,3-bdc system we introduce was discovered by noting that were able to synthesise MOX-2 in ethyl acetate. Repeating this synthesis using the substituted 1,3-bdc analogues 5-X-1,3-bdc (X = Br, Cl, I, F, CH_3_ and NO_2_), we obtained single-crystal products of a common MOF architecture for X = Br, Cl, I, and powder samples for X = F, CH_3_ and NO_2_. The MOX-4 structure type, determined from the X = Br, Cl, I single crystals, turns out to have been reported previously for other substituted 1,3-bdc-containing MOFs, albeit using very bulky substituents.^25–30^

The structure of MOX-4, which adopts the unusual **ptr** topology, is characterised by hexagonal channels ∼11.1 Å in diameter running parallel to the crystallographic *c* axis [Fig. 4(c)]. These hexagonal channels are particularly striking as their shape and size is comparable to that of the channels found in other porous MOFs, such as MIL-88B (*viz.* ∼11.0 Å)^31^ and MOF-74 (*viz.* ∼15.2 Å).^32^ It is these hexagonal pores that make MIL-88B and MOF-74 well-suited for applications such as gas sorption and catalysis.^33,34^

Similar to MOF-74, the presence of a functional group attached to the 1,3-bdc linker benzene ring forces the framework of MOX-4(X) to be rigid, rather than flexible (as in MIL-88B).^31^ In fact, the interactions between these functional groups appear to be antipolar, in the sense that for each pore lined by six 5-X-1,3-bdc linkers, three are pointing up along the channel, and three are pointing down. For each linker that points up, its two neighbours point down. These configurations are consistent along the same columns—*i.e.* all linkers stacked along the *c* crystallographic axis point in the same way along the channel. Since the distance between neighbouring linkers within the same layer is ∼6.8 Å, it is likely that these antipolar interactions are governed by steric hindrance.

Additionally, each linker interacts with another linker from a neighbouring channel in a stacked configuration. Again, these interactions are antipolar: if one linker in the pair points down, the other will point up. With a distance of 3.5 Å, the stacked linkers are even closer together than the six linkers lining the channel. Therefore, steric hindrance appears to govern the observed arrangement. Since the lattice of MOX-4 can accommodate these interactions without any geometric frustration, its structure is ordered. Therefore, the functional groups appear to play an important role in directing the structure of MOX-4, likely governed by steric interactions.

MOX-4 has one crystallographically unique 5-X-1,3-bdc linker, although the torsion angles *ϕ*_1_ and *ϕ*_2_ differ as a function of functional group X [Table S4†]. The crystallographic symmetry (*R*3̄*m*) is such that *ϕ*_1_ = *ϕ*_2_, and hence Δ*ϕ* = 0 for all these materials. Additionally, the torsion angles are very small, varying in a nontrivial way as the size of the functional group changes. In the case of X = Cl, the torsion angles are as small as 0.859°.

### 2.3 Linker torsion angles in Zn-(5-X-)1,3-bdc and (2-X-)1,4-bdc phase space

Returning now to the primary goal of our study—namely to understand the similarities and differences between torsional degrees of freedom in TRUMOF-1 and those of related crystalline phases—we assembled a large structural database of torsion angles in crystalline Zn/1,3-bdc MOFs. We included in this database the various MOX-2, MOX-3, and MOX-4(X) structures described above. We also searched the CCDC database for Zn/1,3-bdc MOFs and extracted the linker torsion angles and corresponding *syn*/*anti* phase using the software ConQuest.^35^ We confined our search criteria to identify MOFs containing only a single metal type and a single linker (*i.e.* Zn and 1,3-bdc, respectively). These results include MOFs in which the carboxylates are not all coordinated and/or Zn has a non-tetrahedral coordination environment. We also extended the search to include linkers of the form *n*-X-1,3-bdc with X = Br, Cl, I, F, CH_3_, OCH_3_, OH and NH_2_ for *n* = 2, 4, and 5, obtaining hits only for *n* = 5.

**Fig. 5:**
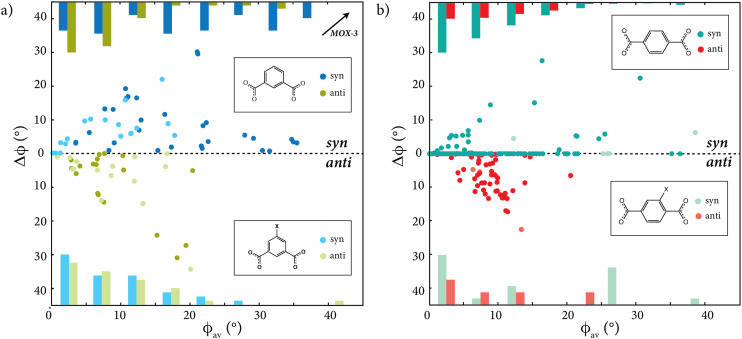
Distribution of (5-X-)1,3-bdc and (2-X-)1,4-bdc torsion angles in crystalline Zn/bdc MOFs. (a) Plot showing the average torsion angle value, *ϕ*_av_, of each (5-X-)1,3-BDC linker carboxylate extracted from all Zn-(5-X-)1,3-BDC MOFs found in the literature. To demonstrate the independence of the two torsion angles within each 1,3-bdc linker, the difference between the two magnitudes, Δ*ϕ*, is given for each torsion angle. Linkers with *syn* and *anti* conformations are plotted above and below the dotted line, respectively. Torsion angle values are coloured according to whether the 1,3-bdc linker is substituted (light colours) or not (dark colours). Histograms are given at the top (1,3-bdc) and bottom (5-X-1,3-bdc) to demonstrate the ratio of *syn* : *anti* conformations for each 5° interval. (b) Plot showing the equivalent data for (2-X-)1,4-bdc linkers extracted from all Zn-(2-X-)1,4-bdc MOFs in our database. Again, torsion angle values are coloured according to whether the 1,4-bdc linker is substituted (light colours) or unsubstituted (dark colours).

Our results are shown in Fig. 5(a), where we use the same representation employed for TRUMOF-1 in Fig. 3. There are significantly fewer data points in this crystalline 1,3-bdc torsion map because crystallographic symmetry constrains the number of inequivalent angles in any one structure—in contrast to the effect of the absence of crystallographic symmetry on the diversity of torsion angles in TRUMOF-1. Nevertheless the first striking result is that—despite the data of Fig. 5(a) coming from experiment and those of Fig. 3(a) from DFT—the *ϕ*_av_ and Δ*ϕ* angles in crystalline Zn/1,3-bdc MOFs nonetheless span much the same values as in TRUMOF-1. In other words, there is nothing particularly unusual about the range of torsion angles in that disordered structure. This finding is a first key result of our study. In fact, it is the crystalline material MOX-3 that shows the most extreme torsional deformation (so much so that it is located off the bounds of our plot).

We make a few additional comments regarding the distribution of torsion angles in crystalline Zn/1,3-bdc frameworks. First, we note that the span of torsion angles is not particularly different for substituted and unsubstituted 1,3-bdc linkers. Second, there is a slight preference for the *syn* configuration for (unsubstituted) 1,3-bdc, whereas the *syn* : *anti* ratio for 5-X-1,3-bdc is close to 1 : 1 [Table S5†]. Third, 1,3-bdc linkers with the *anti* configuration tend to adopt torsion angles less than 10°, whilst those of the *syn* conformation appear capable of adopting a range of both small and large torsion angles—the distribution of *syn* linker torsion angles being essentially even across the 0–40° range. And, fourth, for substituted 5-X-1,3-bdc linkers, both linker conformations gradually decline in frequency with increasing torsion angle.

Having established that TRUMOF-1 samples essentially the same torsional degrees of freedom as crystalline Zn/1,3-bdc MOFs, we turn finally to question the role of the linker itself. Using a similar approach described above, we assembled a database of Zn/1,4-bdc MOFs, again including substituted 1,4-bdc linkers (2-X-1,4-bdc with X = Br, Cl, I, F, CH_3_, OCH_3_, OH and NH_2_).

Our results are shown in Fig. 5(b), and we now observe a number of meaningful differences to the various Zn/1,3-bdc frameworks we have already discussed. For example, both substituted and unsubstituted Zn/1,4-bdc frameworks show a significant preference for the *syn* conformation [Table S5†]. Also, the average torsion angle magnitude is much lower than for Zn/1,3-bdc systems, consistent with the larger energy penalty associated with torsional degrees of freedom in 1,4-disubstituted benzenes. The higher symmetry of 1,4-bdc linkers clearly plays an important role: the majority of such linkers in the *syn* conformation lie on the horizontal axis, with Δ*ϕ* = 0. In these conformations, crystallographic symmetry constrains the two carboxylate substituents to lie in the same plane, with the central benzene ring twisting out of this plane. As such, the mirror symmetry of the 1,4-bdc linker is maintained. The spread of *anti* configured linkers is similar to the torsion angle distribution of 1,3-bdc and 5-X-1,3-bdc, although our certainty here is low as these conformations arise in a relatively small fraction of our database. Finally, the relative population of both linker conformations gradually declines with increasing torsion angle.

## 3 Concluding remarks

So, from a crystal engineering perspective, our analysis has shown that the torsional degrees of freedom in the topologically aperiodic TRUMOF-1 are not meaningfully different to those observed in crystalline Zn/1,3-bdc MOFs, but that the higher-symmetry 1,4-bdc linker places strong constraints on the torsional flexibility of many Zn/1,4-bdc MOFs. The former observation hints at why the TRUMOF-1 topology is even accessible—the configurational landscape being sufficiently shallow—and the latter suggesting why 1,4-bdc favours high-symmetry crystalline structures. Indeed it is striking from our maps that the two torsion angles of 1,3-bdc linkers are essentially independent in nearly all Zn/1,3-bdc MOFs, whether crystalline or not. Likewise, *syn* and *anti* conformations are equally likely. The same is not true for the 1,4-bdc systems, for which the two carboxylates tend to lie in the same plane and a *syn* conformation is strongly preferred.

The independent torsion angle behaviour of the two carboxylate moieties in 1,3-bdc may also explain the difficulty of applying reticular chemistry methodologies within the Zn/1,3-bdc phase space. In the case of 1,4-bdc MOFs, the additional steric bulk caused by substitution of the central benzene ring can be accommodated by a twist of the ring, without breaking the symmetry that relates one carboxylate torsion angle to the other. This phenomenon is observed for an **nbo** structure-type Cu-MOF, for example, in which Br functionalised 1,4-bdc induces the 90°-twist responsible for the topology.^36^ The benzene ring in 1,3-bdc cannot perform a full 180° rotation, however, and therefore compensates for the added bulkiness through crystallising in a different topology as permitted by the independent torsional flexibility of the two carboxylates. This proposed mechanism is demonstrated with the denser frameworks TRUMOF-1 and MOX-2. In both cases there is insufficient room to accommodate additional substituents on the 1,3-bdc linker. Instead, derivatives substituted at either the 5- or 4,6-positions of the ring resulted in MOX-4 and MOX-3, respectively. The former has very large pores lined by the functional groups, and the structure of the latter consists of layers held together by non-covalent interactions between the separated functional groups. In this way, the functional group appears to have a significant structure-directing role, driven by repulsive and/or interactive interactions.

In turn, the rather rigid 1,4-bdc linker facilitates crystallisation of Zn-MOFs—in contrast to 1,3-bdc, which behaves in a more flexible way. We suggest that this is why synthetic parameters have to be particularly well defined to identify the right conditions for crystallisation of Zn/1,3-bdc MOFs. Perhaps as a result, ConQuest finds a lower number of reported Zn/1,3-bdc MOFs from the literature (50) compared to Zn/1,4-bdc (189).

Our study has intentionally focussed on Zn^2+^-containing MOFs, but at face value we expect the general trends observed here to translate to benzenedicarboxylate frameworks assembled using other transition metals. Variation in the transition-metal size and charge may affect coordination numbers and the degree of covalency in the metal–ligand interaction, which will in turn modify the degree of structural flexibility around the transition-metal centre itself. But we expect that any effect on the torsional degrees of freedom of the linkers will be minimal. We do not yet know, however, whether analogues of TRUMOF-1 containing transition-metals other than Zn are synthetically accessible.

To conclude, our study demonstrates that, in addition to introducing orientational degrees of freedom, the lower symmetry of 1,3-bdc leads to a conformational flexibility in the independent torsional motion of the carboxylates. Both of these properties contribute to the observed complexity of the Zn/1,3-bdc phase space, and are likely relevant to the accessibility of an aperiodic material such as TRUMOF-1. As such, the approach of incorporating low-symmetry linkers offers an efficient general methodology for obtaining complex MOF phases, as discussed in detail elsewhere.^37^ However, there is not yet any clearly systematic way of obtaining a topologically aperiodic phase such as TRUMOF-1. We have shown that the synthetic parameters required to obtain TRUMOF-1 are very specific—any alteration to the solvent or reaction time resulted in a different phase or crystals of lesser quality. This observation further reflects the shallow energy landscape of the Zn/1,3-bdc system and indicates that, in order to obtain additional TRUMOFs, a great deal of trial-and-error may be required. This challenge of finding suitable synthetic parameters, together with addressing the ultimate goal of controlling disorder and thus the aperiodic connectivity, are important aspects on which to focus in future research.

## Conflicts of interest

There are no conflicts to declare.

## Supplementary Material

CE-026-D3CE01078C-s001

CE-026-D3CE01078C-s002
