# Supplementary material for: Torsional flexibility in zinc–benzenedicarboxylate metal–organic frameworks
Source: CrystEngComm. 2023 Dec 29;26(5):673–80. doi: 10.1039/d3ce01078c (PMC10823780; doi:10.1039/d3ce01078c)
Supplement: CE-026-D3CE01078C-s001 [file CE-026-D3CE01078C-s001.pdf]

Electronic Supplementary Material (ESI) for CrystEngComm.
This journal is © The Royal Society of Chemistry 2024

Torsional flexibility in zinc–benzenedicarboxylate metal–organic frameworks†

SUPPLEMENTARY INFORMATION

Emily G. Meekel,[a] Thomas C. Nicholas,[a] Ben Slater,[b] and Andrew L. Goodwin[a,*]

[a]Department of Chemistry, University of Oxford, Inorganic Chemistry Laboratory,
South Parks Road, Oxford OX1 3QR, U.K.
[b]Department of Chemistry, University College London,
20 Gordon Street, London WC1H 0AJ, U.K.
*To whom correspondence should be addressed; E-mail: andrew.goodwin@chem.ox.ac.uk.

Submitted to CrystEngComm

1 Geometry optimisation

Full cell optimisations were carried out using the Quickstep module of the CP2K package[S1]. We used the DZVP MOLOPT basis sets for all atoms, the PBE functional[S2] and the “D3” van der Waals correction scheme[S3]. A 800 Ry cut-off was used in conjunction with a reference cell for smooth optimisation of the cell parameters and atomic coordinates until the forces on the atoms were less than 0.6 kJ mol^{-1} Å^{-1}. No atomic constraints were applied during the relaxation and no symmetry was imposed upon the cell parameters or coordinates (in effect the cells are relaxed in $P1$).

See Ref. 4 for a more detailed outline of the DFT work, including summaries of the optimised cell parameters and relative energies.

2 Linker torsion angles

TRUMOF-1

Syn(%)	Anti(%)	Total (100%)
44	56	2,134

Table S1: Syn:anti ratio of 1,3-bdc linkers in TRUMOF-1.

MOX-2

ϕ_1 / °	ϕ_2 / °	syn/anti
17.87	12.269	syn
9.8425	33.61	syn
23.6525	23.6525	syn

Table S2: Torsion angles of three unique 1,3-bdc linkers found in MOX-2

MOX-3

ϕ_1 / °	ϕ_2 / °	syn/anti
30.25	83.56	syn

Table S3: Torsion angles of 1,3-bdc linker found in MOX-3

MOX-4

X	$\phi_{1,2}$ / °	syn/anti
Br	1.821	syn
Cl	0.859	syn
I	1.929	syn

Table S4: Torsion angles of 1,3-bdc linker found in MOX-4

Zn/1,3-bdc and 1,4-bdc MOF phase space

	Syn(%)	Anti(%)	Total (100%)
1,3-bdc	60	40	50
1,4-bdc	72	28	189
1,3-bdc-X	51.6	48.4	31
1,4-bdc-X	66.7	33.3	15

Table S5: Syn:anti ratio's of 1,3-bdc and 1,4-bdc incorporated MOFs.

Influence porosity/density

To investigate whether the variety in linker torsion angle may have an influence on the porosity of TRUMOF-1, the data presented in Fig. 3a in the main text was plotted with a colour gradient correlating to the density of the supercell (Fig. S1a). In addition, similar to Fig. 3b in the main text, the average torsion angle of ϕ_1 and ϕ_2 for each supercell was plotted against the density of each supercell (Fig. S1b). Both figures show no correlation between the density and torsion angle distribution of TRUMOF-1, indicating the degree of linker torsion angle has no significant influence on the porosity.

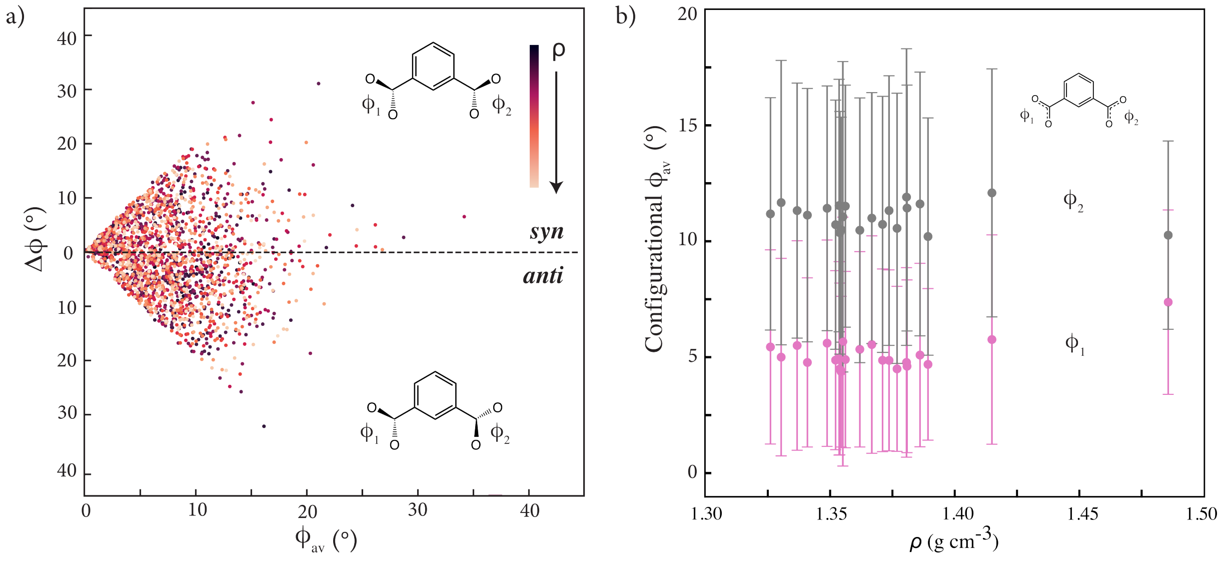

Figure S1: Distribution of 1,3-bdc torsion angle values observed in TRUMOF-1 DFT supercell configurations. (a) Plot showing the distribution of both average torsion angle magnitude, ϕ_{av}, and difference in torsion angle magnitudes $\Delta\phi$ within each 1,3-bdc linker in the various supercells. Linkers with *syn* and *anti* conformations are plotted above and below the dotted line, respectively. All torsion angle values are coloured according to the density of the supercell to which they correspond. (b) Graph showing the average values of ϕ_1 (pink) and ϕ_2 (grey) for each supercell as a function of its density. Note that in order to calculate the average value of ϕ_1 and ϕ_2, the carboxylate with the largest torsion angle was assigned ϕ_1, leaving the smaller torsion angle to be assigned ϕ_2. The standard deviation in torsion angle is represented with error bars.

3 Synthesis

MOX-2

MOX-2 was synthesised by combining solutions of $Zn(NO_3)_2 \cdot 6\ H_2O$ (0.33 mmol) and 1,3-bdc (0.33 mmol) in EA (5 mL). The resulting mixture was placed in a Teflon chamber and sealed, which in turn was fitted in an autoclave. The Teflon lined autoclave was placed in an oven and heated to 110 °C. After 14 h at this temperature, the system was slowly cooled to room temperature to afford MOX-2 as colourless, single-crystal rods amidst intergrown clusters. The crystals were isolated by filtration and washed three times with EA. Each washing cycle involved soaking MOX-2 crystals in fresh EA for 24 h, followed by filtration.

MOX-3

MOX-3 was synthesised by dissolving 0.33 mmol (97.7 mg) $Zn(NO_3)_2 \cdot 6\ H_2O$ and 0.33 mmol (106.9 mg) 4,6-dibromo isophthalic acid (4,6-Br_2-1,3-bdc) in DMF (5 mL). The resulting solution was then heated to 110 °C in a sealed vial for 14 h resulting in clear, colourless, hexagonal shaped disks (Fig. S2).

By placing the disks in paratone-n oil and leaving them for *ca.* 2 h, the hexagonal disks turned into oval-shaped crystals (Fig. S2). These crystals belong to the phase of MOX-3. They were kept in oil and therefore not washed nor treated with other solvents for analysis.

MOX-4

MOX-4 was synthesised by combining solutions of $Zn(NO_3)_2 \cdot 6\ H_2O$ (0.33 mmol) and 5-X-1,3-bdc (0.33 mmol) in EA (5 mL) for X = Br, Cl, I, F, NO_2, CH_3, OH, Br/Cl (1:1) and Br/H (1:1). The resulting mixture was placed in a Teflon chamber and sealed, which in turn was fitted in an autoclave. The Teflon lined autoclave was placed in an oven and heated to 110 °C. After 14 h at this temperature, the system was cooled to room temperature to afford MOX-3 as colourless crystals, most of which intergrown clusters, though some single crystals (Fig. 3.8a). The crystals were isolated by filtration and washed three times with EA. Each washing cycle involved soaking MOX-4 crystals in fresh EA for 24 h, followed by filtration.

Single crystals were obtained for X = Br, Cl, I, and Br/Cl, powders were obtained for X = F, NO_2, CH_3 and Br/H. For X = OH, no product was obtained.

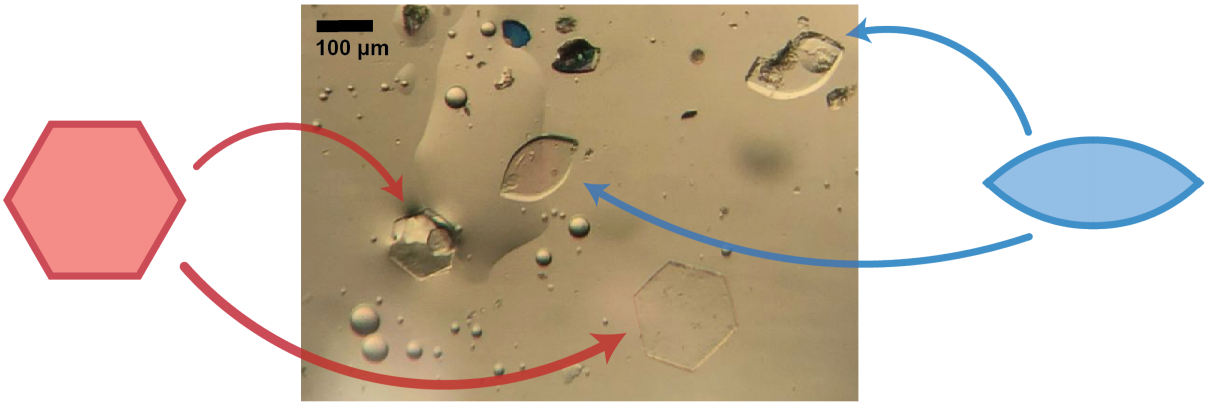

Figure S2: Batch of MOX-5 crystals, indicating both oval- and hexagonal-shaped crystals with blue and red arrows, respectively. Scale bar given in the top left corner for reference (100 μm)

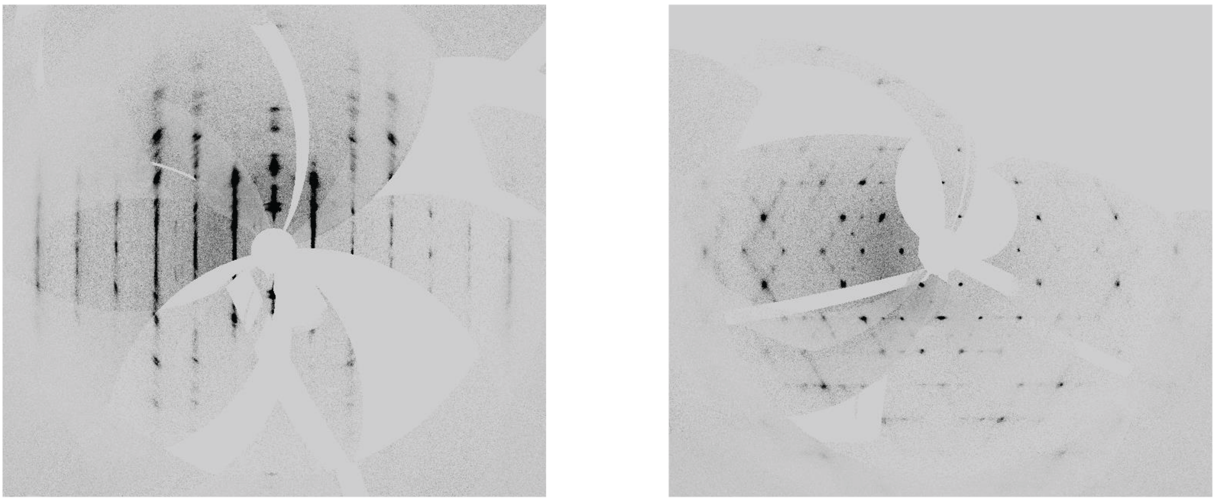

Figure S3: Reconstructed "0*kl*" (left) and "*hk*0" (right) layers of MOX-5α.

4 Crystallographic refinements

MOX-2

The single crystal diffraction pattern of MOX-2 was measured using a Rigaku Synergy S diffractometer fitted with a Dectris EIGER2 R 1M detector. The instrument outputted mirror-monochromated Cu-Kα (λ = 1.5406 Å) and was equipped with an Oxford Cryosystems Cryostream 700 for temperature control.

A washed crystal of MOX-2 was isolated directly with EA, dried in air, and then mounted onto a 0.2 mm diameter MiTeGen loop using Paratone-N oil as a cryoprotectant. The crystal was then attached to the instrument goniometer before data were collected about ϕ and ω rotations.

Unit cell determination, data integration, frame scaling, and absorption corrections (multi-scan implemented in ABSPACK) with beam profile correction) were performed in CrysAlisPro[S5]. A structure solution was obtained using intrinsic phasing methods from ShelXT[S6], followed with refinements using a full-matrix least-squares approach on all unique F^2 values. The refinements were performed using ShelXL[S7] as implemented within Olex2-1.5[S8].

An initial solution in the non-centrosymmetric orthorhombic space group $P2_12_12_1$ with a = 11.0562(2) Å, b = 12.6155(2) Å, and c = 15.2102(2) Å showed clearly the presence of two Zn atoms directly connected to 1,3-bdc, forming distorted ZnO_4 tetrahedra. The remaining electron density was found to originate from ordered solvent in the pores. All non-hydrogen atoms could be treated by an unrestrained anisotropic refinement unless otherwise stated. The H-atom positions were added in their calculated position and refined using riding thermal parameters.

Final structural parameters are given in Table S6, with the asymmetric unit shown in Fig. S4. The structure has been deposited with the Cambridge crystallographic data centre (CCDC) under the reference number 2302224.

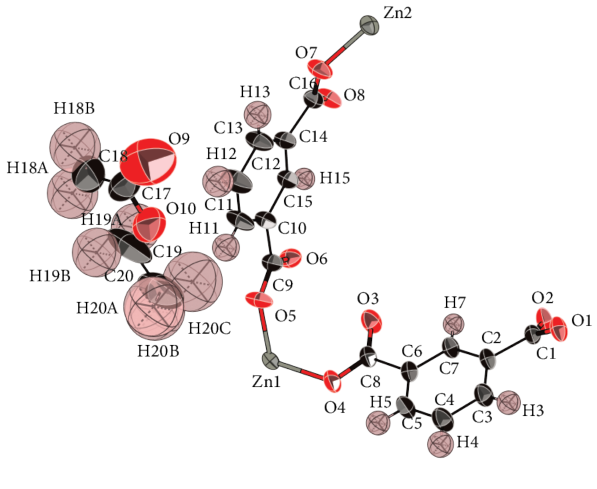

Figure S4: Asymmetric unit of MOX-2 at 298 K. Ellipsoids are drawn at 50% probability. Colour scheme: C atoms are shown in black, O atoms in red, Zn atoms in grey and H atoms in pink.

Table S6: Crystal data and structure refinement for MOX-2.

Empirical formula	$C_{10}H_8O_5$ Zn
Formula weight / g mol^{-1}	273.53
Temperature / K	295.15
Crystal system	orthorhombic
Space group	$P2_12_12_1$
a / Å	11.0562(2)
b / Å	12.6155(2)
c / Å	15.2102(3)
α / °	90
V / Å^3	2121.51(7)
Z	8
ρ_{calc} / g cm^{-3}	1.713
μ / mm^{-1}	2.316
F(000)	1104.0
Crystal size / mm^3	0.18 $\times$ 0.40 $\times$ 0.40
Radiation	CuKα (λ = 1.54184 Å)
2θ range for data collection / °	6.502 – 61.046
Index ranges	-15 $\leq h \leq 13, -18 \leq k \leq 16, -21 \leq l \leq 19$
Reflections collected	35121
Independent reflections	5669[R$_{int}$=0.0431, R$_{sigma}$=0.0327]
Data/restraints/parameters	5669/0/291
Goodness-of-fit on F^2	1.056
Final R indexes [I>2sigma(I)]	R$_1$ = 0.0431, wR$_2$ = 0.0327
Final R indexes [all data]	R$_1$ = 0.0392, wR$_2$ = 0.0761
Largest diff. peak/hole / e Å^{-3}	0.43/–0.42
Flack parameter	–0.005(6)

Powder X-ray diffraction

To verify the phase purity of the MOX-2 sample, a powder X-ray diffraction experiment was performed at ambient conditions using a high-intensity Bruker D8 Advance Eco diffractometer equipped with a Cu-Kα source (λ = 1.54 Å) and a fluorescence-filtering LYNXEYE XE-T detector.

Pawley refinements were performed on the PXRD data using the TOPAS-ACADEMIC software[S9] (Fig. S5). This resulted in a reasonable fit (R_{wp} = 12.9%) using a refinement model containing a single phase with $P2_12_12_1$ space-group symmetry; the refined lattice parameter obtained was a = 11.068(9) Å, b = 12.835(4) Å, and c = 15.281(8) Å.

The observed additional phases likely arise from unreacted starting material; both the $Zn(NO_3)_2 \cdot H_2O$ salt and 1,3-bdc linker did not dissolve in the EA solvent at room temperature. Whilst heating in the oven, the solution may become saturated, in turn leaving some of the starting material undissolved.

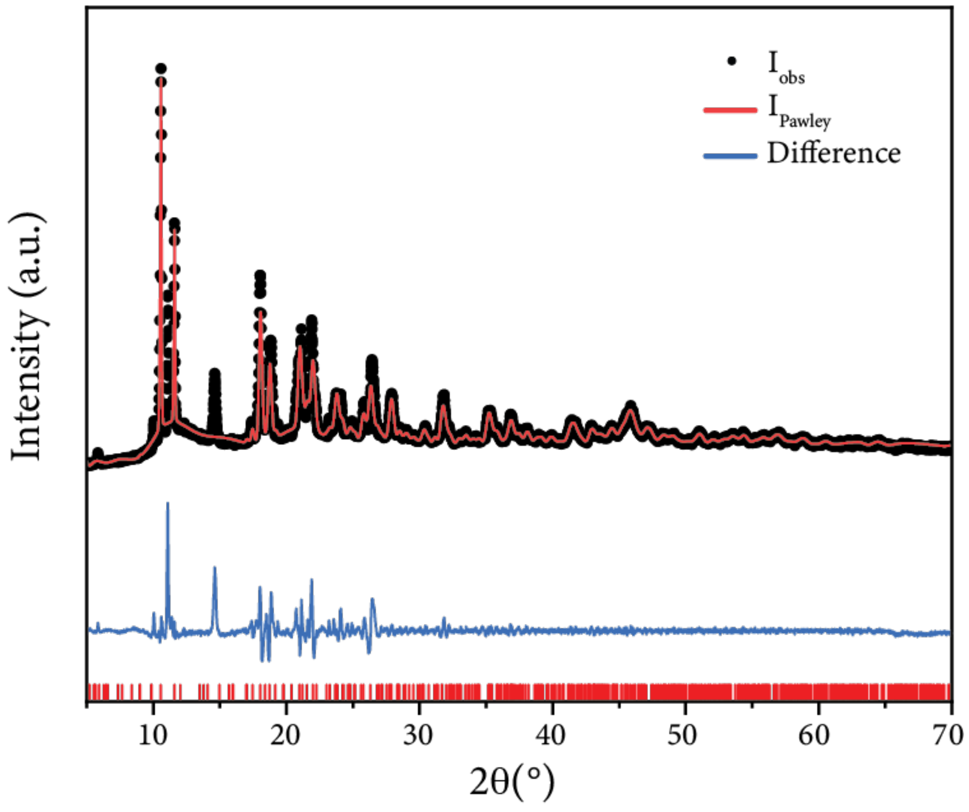

Figure S5: Pawley refinement of the experimental MOX-2 PXRD pattern, resulting in a = 11.068(9) Å, b = 12.835(4) Å and c = 15.281(8) Å for $P2_12_12_1$.

MOX-3

The SCXRD patterns of both crystals were measured using a Rigaku Synergy S diffractometer fitted with a Dectris EIGER2 R 1M detector. The instrument outputted mirror-monochromated Cu-Kα (λ = 1.5406 $\mathring{A}$) and was equipped with an Oxford Cryosystems Cryostream 700 for temperature control.

Crystals of both MOX-3α and MOX-5 were isolated directly with DMF and mounted onto a 0.2 mm diameter MiTeGen loop using Paratone-N oil as a cryoprotectant. The crystals were then attached to the instrument goniometer before data were collected about ϕ and ω rotations.

Unit cell determination, data integration, frame scaling, and absorption corrections (multi-scan implemented in ABSPACK) were performed in CrysAlisPro[S5]. A structure solution was obtained using intrinsic phasing methods from ShelXT[S6], followed with refinements using a full-matrix least-squares approach on all unique F^2 values. The refinements were performed using ShelXL[S7] as implemented within Olex2-1.5[S8].

For the crystalline phase of MOX-3, an initial solution in the centrosymmetric monoclinic space group $P2_1/n$ (no. 14) with a = 12.7970(2) Å, b = 9.5085(1) Å, c = 16.9103(2) Å, and β = 111.969(2)° showed clearly the presence of a Zn atom on the $4e$ Wyckoff position directly connected to a 4,6-Br_2-1,3-bdc linker, forming distorted ZnO_4 tetrahedra. The remaining electron density was found to originate from ordered DMF solvent, of which the O atom is coordinated to the Zn atom. All non-H atoms could be treated by an unrestrained anisotropic refinement unless otherwise stated. The H-atom positions were added in their calculated position and refined using riding thermal parameters.

Final structural parameters are given in Table S7, with the asymmetric unit shown in Fig. S6. The structure has been deposited with the Cambridge crystallographic data centre (CCDC) under the reference number 2302220.

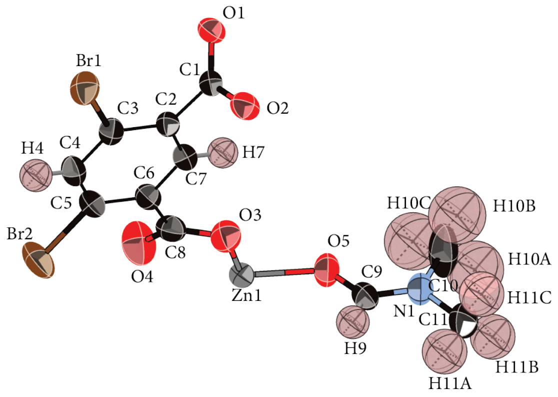

Figure S6: Asymmetric unit of MOX-3 at 298 K. Ellipsoids are drawn at 50% probability. Colour scheme is as follows: C = black spheres, O = red spheres, N = blue spheres, Zn = grey spheres, Br = brown spheres and H = pink spheres.

Table S7: Crystal data and structure refinement for MOX-5.

Empirical formula	$C_{11}H_9O_5NZn$
Formula weight / g mol^{-1}	460.38
Temperature / K	300.15
Crystal system	monoclinic
Space group	$P2_1/n$
a / Å	12.8068(2)
b / Å	9.51590(10)
c / Å	16.9226(2)
α / °	90
β / °	111.994(2)
γ / °	90
V / Å^3	1912.24(5)
Z	4
ρ_{calc} / g cm^{-3}	1.599
μ / mm^{-1}	6.815
F(000)	888.0
Crystal size / mm^3	0.05 $\times$ 0.40 $\times$ 0.70
Radiation	CuKα (λ = 1.54184 Å)
2θ range for data collection / °	7.466 – 152.136
Index ranges	-16 $\leq h \leq 16, -11 \leq k \leq 11, -21 \leq l \leq 20$
Reflections collected	15253
Independent reflections	3947[R_{int}=0.0299, R_{sigma}=0.0277]
Data/restraints/parameters	3947/0/183
Goodness-of-fit on F^2	1.117
Final R indexes [I>2sigma(I)]	R_1 = 0.0300, wR_2 = 0.0898
Final R indexes [all data]	R_1 = 0.0327, wR_2 = 0.0917
Largest diff. peak/hole / e Å^{-3}	0.50/–0.57

Powder X-ray diffraction

Due to both the low quality and instability of the MOX-3 crystals, it was not possible to perform PXRD to confirm the phase purity of the sample.

MOX-4

All MOX-4 single crystal diffraction patterns were measured using a Rigaku Synergy S diffractometer fitted with a Dectris EIGER2 R 1M detector. The instrument produced mirror-monochromated Cu-Kα (λ = 1.5406 Å) and was equipped with an Oxford Cryosystems Cryostream 700 for temperature control.

A washed crystal of MOX-4 was isolated directly with EA, dried in air, and then mounted onto a 0.2 mm diameter MiTeGen loop using Paratone-N oil as a cryoprotectant. The crystal was then attached to the instrument goniometer before data were collected about ϕ and ω rotations, using an exposure time of 0.1 s.

Unit cell determination, data integration, frame scaling, and absorption corrections (multi-scan implemented in ABSPACK) were performed in CrysAlisPro[S5]. A structure solution was obtained using intrinsic phasing methods from ShelXT[S6], followed with refinements using a full-matrix least-squares approach on all unique F^2 values. The refinements were performed using ShelXL[S7] as implemented within Olex2-1.5[S8].

An initial solution in the non-centrosymmetric rhombohedral space group $R\bar{3}m$ (lattice parameters given in Table 3.1) showed clearly the presence of distorted ZnO_4 tetrahedra centred on the 18*f* Wyckoff position, connected by 5-X-1,3-bdc linkers. The remaining electron density was determined to originate from disordered EA solvent molecules in the pores. Their exact positions and orientation was not able to be solved. Since the remaining electron density was quite low, no solvent masking was used to account for this.

All non-hydrogen atoms could be treated by an unrestrained anisotropic refinement unless otherwise stated. The H-atom positions were added in their calculated position and refined using riding thermal parameters.

Final structural parameters are given in Tables S8, S9, S10, and S11, with the asymmetric unit shown in Fig. S7. The structures have been deposited with the Cambridge crystallographic data centre (CCDC) under the reference numbers 2302221–2302223, and 2302225.

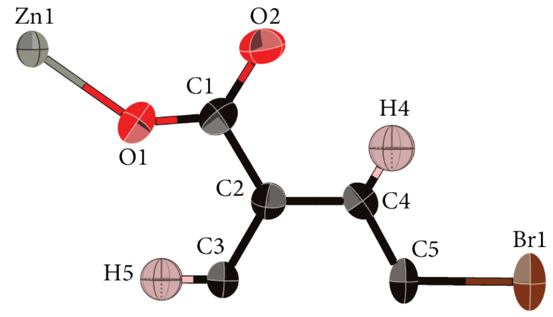

Figure S7: Asymmetric unit of MOX-4 at 298 K. Ellipsoids are drawn at 50% probability. Colour scheme is as follows: C = black spheres, O = red spheres, Zn = grey spheres and H = pink spheres.

Table S8: Crystal data and structure refinement for MOX-4 for X = Br.

Empirical formula	$C_8H_3O_4$ZnBr
Formula weight / g mol^{-1}	309.39
Temperature / K	293
Crystal system	trigonal
Space group	$R\bar{3}m$
a / Å	28.4852(3)
b / Å	28.4852(3)
c / Å	7.90520(10)
α / °	90
β / °	90
γ / °	120
V / Å^3	5554.97(14)
Z	18
ρ_{calc} / g cm^{-3}	2.127
μ / mm^{-1}	8.292
F(000)	3427.0
Crystal size / mm^3	0.10 × 0.15 × 0.60
Radiation	CuKα (λ = 1.54184 Å)
2θ range for data collection / °	10.758 – 151.982
Index ranges	-35 $\leq h \leq 35, -35 \leq k \leq 34, -7 \leq l \leq 9$
Reflections collected	21620
Independent reflections	1372[R_{int}=0.0644, R_{sigma}=0.068]
Data/restraints/parameters	1372/9/70
Goodness-of-fit on F^2	1.133
Final R indexes [I>2sigma(I)]	R_1 = 0.0613, wR_2 = 0.1998
Final R indexes [all data]	R_1 = 0.0622, wR_2 = 0.2010
Largest diff. peak/hole / e Å^{-3}	1.90/–1.70

Table S9: Crystal data and structure refinement for MOX-4 for X = Cl.

Empirical formula	$C_8H_3O_4ZnCl$
Formula weight / g mol^{-1}	263.92
Temperature / K	295.15
Crystal system	trigonal
Space group	$R\bar{3}m$
a / Å	28.4445(5)
b / Å	28.4445(5)
c / Å	7.82240(10)
α / °	90
β / °	90
γ / °	120
V / Å^3	5481.1(2)
Z	18
ρ_{calc} / g cm^{-3}	1.439
μ / mm^{-1}	4.751
F(000)	2340.0
Crystal size / mm^3	0.10 × 0.15 × 0.60
Radiation	CuKα (λ = 1.54184 Å)
2θ range for data collection / °	10.774 – 151.7
Index ranges	-35 $\leq h \leq 35, -33 \leq k \leq 35, -9 \leq l \leq 9$
Reflections collected	24893
Independent reflections	1357[R_{int}=0.0531, R_{sigma}=0.0160]
Data/restraints/parameters	1357/6/69
Goodness-of-fit on F^2	1.124
Final R indexes [I>2sigma(I)]	R_1 = 0.0492, wR_2 = 0.1553
Final R indexes [all data]	R_1 = 0.0515, wR_2 = 0.1576
Largest diff. peak/hole / e Å^{-3}	0.94/–0.77

Table S10: Crystal data and structure refinement for MOX-4 for X = Cl/Br.

Empirical formula	$C_8H_3O_4ZnBr_{0.4}Cl_{0.6}$
Formula weight / g mol^{-1}	282.05
Temperature / K	295.01
Crystal system	trigonal
Space group	$R\bar{3}m$
a / Å	28.4902(4)
b / Å	28.4902(4)
c / Å	7.90730(10)
α / °	90
β / °	90
γ / °	120
V / Å^3	5558.40(17)
Z	18
ρ_{calc} / g cm^{-3}	1.517
μ / mm^{-1}	5.419
F(000)	2472.0
Crystal size / mm^3	0.10 $\times$ 0.15 $\times$ 0.60
Radiation	CuKα (λ = 1.54184 Å)
2θ range for data collection / °	10.758 – 151.228
Index ranges	-34 $\leq h \leq 34, -34 \leq k \leq 26, -9 \leq l \leq 9$
Reflections collected	7373
Independent reflections	1362[R_{int}=0.0330, R_{sigma}=0.0201]
Data/restraints/parameters	1362/0/70
Goodness-of-fit on F^2	1.103
Final R indexes [I>2sigma(I)]	R_1 = 0.0541, wR_2 = 0.1769
Final R indexes [all data]	R_1 = 0.0560, wR_2 = 0.1792
Largest diff. peak/hole / e Å^{-3}	1.70/–1.14

Table S11: Crystal data and structure refinement for MOX-4 for X = I.

Empirical formula	$C_8H_3O_4ZnI$
Formula weight / g mol^{-1}	355.37
Temperature / K	290.15
Crystal system	trigonal
Space group	$R\bar{3}m$
a / Å	28.5799(4)
b / Å	28.5799(4)
c / Å	8.09130(10)
α / °	90
β / °	90
γ / °	120
V / Å^3	5723.61(18)
Z	18
ρ_{calc} / g cm^{-3}	1.856
μ / mm^{-1}	21.677
F(000)	2988.0
Crystal size / mm^3	0.20 $\times$ 0.20 $\times$ 0.60
Radiation	CuKα (λ = 1.54184 Å)
2θ range for data collection / °	10.724– 151.978
Index ranges	-35 $\leq h \leq 33, -20 \leq k \leq 35, -10 \leq l \leq 9$
Reflections collected	7511
Independent reflections	1405[R_{int}=0.0194, R_{sigma}=0.0095]
Data/restraints/parameters	1405/8/69
Goodness-of-fit on F^2	1.118
Final R indexes [I>2sigma(I)]	R_1 = 0.0666, wR_2 = 0.2117
Final R indexes [all data]	R_1 = 0.0670, wR_2 = 0.2121
Largest diff. peak/hole / e Å^{-3}	3.26/–2.49

Powder X-ray diffraction

To verify the phase purity of the MOX-4 samples, powder X-ray diffraction experiment were performed at ambient conditions using a high-intensity Bruker D8 Advance Eco diffractometer equipped with a Cu-Kα source (λ = 1.54 Å) and a fluorescence-filtering LYNXEYE XE-T detector. The obtained diffraction patterns were subsequently compared to patterns calculated from the single-crystal models, as the unit cells appeared to large to perform an accurate Pawley fit (Fig. S8).

Interestingly, the phase purity of the samples — as derived from comparison of the experimental and simulated patterns — seems to decrease as the size of the linker functional group becomes smaller. This could imply that the functional group has a structure directing role, ultimately lining the large hexagonal channels and keeping them apart through *e.g.* steric hindrance.

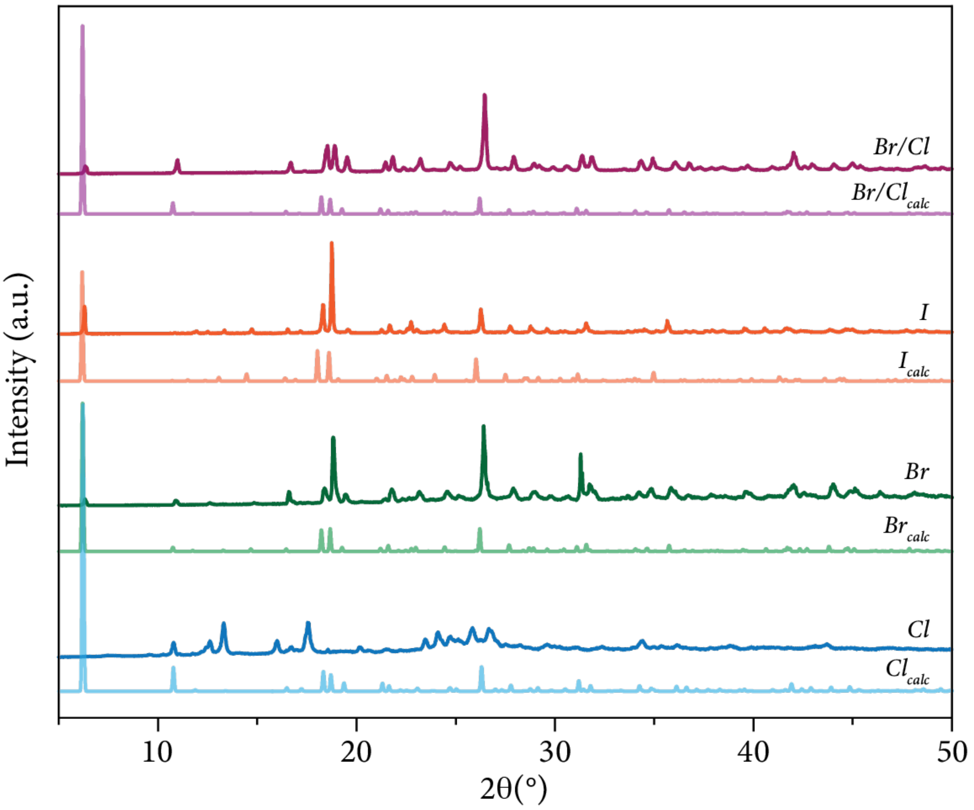

Figure S8: PXRD patterns of all MOX-4 samples (*i.e.* with X = Br/Cl, I, Br and Cl). For all samples, the experimental pattern is given at the top (dark colour), with the simulated pattern given at the bottom (light colour). Each pattern is labelled at the top right corner.

5 References

(S1) T. D. Kühne, M. Iannuzzi, M. Del Ben, V. V. Rybkin, P. Seewald, F. Stein, T. Laino, R. Z. Khaliullin, O. Schütt, F. Schiffmann, D. Golze, J. Wilhelm, S. Chulkov, M. H. Bani-Hashemian, V. Weber, U. Borštnik, M. Taillefumier, A. S. Jakobovits, A. Lazzaro, H. Pabst, T. Müller, R. Schade, M. Guidon, S. Andermatt, N. Holmberg, G. K. Schenter, A. Hehn, A. Bussy, F. Belleflamme, G. Tabacchi, A. Glöß, M. Lass, I. Bethune, C. J. Mundy, C. Plessl, M. Watkins, J. VandeVondele, M. Krack and J. Hutter, *J. Chem. Phys.*, 2020, **152**, 194103.

(S2) J. P. Perdew, K. Burke and M. Ernzerhof, *Phys. Rev. Lett.*, 1996, **77**, 3865–3868.

(S3) S. Grimme, J. Antony, S. Ehrlich and H. Krieg, *J. Chem. Phys.*, 2010, **132**, 154104.

(S4) E. G. Meekel, E. M. Schmidt, L. J. Cameron, A. D. Dharma, H. J. Windsor, S. G. Duyker, A. Minelli, T. Pope, G. O. Lepore, B. Slater, C. J. Kepert and A. L. Goodwin, *Science*, 2023, **379**, 357–361.

(S5) *CrysAlis PRO. Agilent Technologies Ltd, Yarnton, Oxfordshire, England*, 2014.

(S6) G. M. Sheldrick, *Acta Cryst. A*, 2015, **71**, 3–8.

(S7) G. M. Sheldrick, *Acta Cryst. C*, 2015, **71**, 3–8.

(S8) O. V. Dolomanov, L. J. Bourhis, R. J. Gildea, J. A. K. Howard and H. Puschmann, *J. Appl. Cryst.*, 2009, **42**, 339–341.

(S9) A. A. Coelho, *J. Appl. Cryst.*, 2018, **51**, 210–218.
